# Loop Diuretics and Sarcopenia: A Potential Association

**DOI:** 10.3390/muscles2040024

**Published:** 2023-09-22

**Authors:** Nikolaos D. Karakousis, Petros N. Georgakopoulos

**Affiliations:** 1 Independent Researcher, Vrilissia 15235, Greece; 2 Internal Medicine Department, Primary Healthcare, Amarousion 15125, Greece; pegeorgakopoulos@yahoo.gr

**Keywords:** loop diuretics, sarcopenia, liver cirrhosis, chronic kidney disease, heart failure

## Abstract

Background: Loop diuretics (LDs) are used to treat various health conditions including heart failure (HF), liver cirrhosis, and chronic kidney disease (CKD). Sarcopenia is a skeletal muscle health issue related to the depletion and decrease of muscle mass and strength, leading to adverse outcomes including frailty syndrome, functional decline, falls, hospitalizations, augmented length of hospital stay, and increased morbidity and mortality. Methods: This study investigated the probable association between LD use and sarcopenia via conducting a non-systematic review of the existing literature. Results: In subjects with non-dialysis-dependent chronic kidney disease (NDD-CKD), an augmented risk of sarcopenia is significantly associated with LD use. Interestingly, in patients with HF treated with LDs, thigh and arm circumferences were significantly small, which is indicative of skeletal muscle wasting. Additionally, in anorexic subjects who are more likely to be on diuretic medication, suffering also from cachexia, a higher prevalence of sarcopenia was demonstrated. In cirrhotic subjects, the treatment dosage of LDs was inversely correlated with the skeletal muscle area per year (ΔSMA). Nevertheless, in subjects with liver cirrhosis treated with LDs, who were divided into those with and those without muscle cramps, the presence of sarcopenia was similar. Conclusions: Further investigation is imperative to validate potential interplay between LDs and sarcopenia.

## 1. Introduction

Sarcopenia is a progressive and generalized skeletal muscle derangement that can be associated not only with advancing age, but also with various long-term health issues [1]. This clinical condition is already related to many adverse and detrimental outcomes including impaired mobility, functional decline, frailty syndrome, falls, hospitalizations and augmented length of stay, raised healthcare costs, and increased morbidity and mortality [1,2,3].

The concept and term of sarcopenia was initially recorded in 1988 by Irwin Rosenberg and denoted a medical issue of muscle loss that occurs in the old [4]. In 1931, Macdonald Critchley, a neurologist located in London, was the first scientist in the present literature to link the wastage of skeletal muscle to aging, which he did simply by observing that musculature tends to decrease in the old [4].

In 2010, the European Working Group on Sarcopenia in Older People (EWGSOP) published a definition of sarcopenia, which was broadly utilized globally, in which the definition included progress in identifying and caring for subjects at risk for or with sarcopenia [5]. In early 2018, the Working Group met again (EWGSOP2) to examine the need for an upgrade concerning the concept of sarcopenia. To raise awareness and care concerning the concept of sarcopenia, the EWGSOP2 upgraded its definition and strategies related to diagnosis in 2018. The specific goals of the upgrade were to: (i) build a sarcopenia definition that addresses recent progress in the scientific, clinical and epidemiological understanding of skeletal muscle; (ii) demonstrate variables that distinguish sarcopenia and predict outcomes, and determine optimum tools for evaluating each variable; (iii) demonstrate cut-off points for measured variables; and (iv) recommend an upgraded screening and evaluation method that is easy to utilize in everyday clinical practice [5].

The 2018 operational definition of sarcopenia included three criteria [5]. These criteria were: (a) low muscle strength, (b) low muscle quantity or quality, and (c) low physical performance [5]. Potential sarcopenia is identified using criterion (a). Diagnosis is validated via the additional record of criterion (b). Importantly, if criteria (a–c) are all met, sarcopenia is characterized as severe [5]. In order to implement this definition in everyday clinical practice, varied and different tools and tests are frequently utilized to evaluate muscle performance and properties, while an updated algorithm is further used to identify sarcopenia cases, provide sarcopenia diagnose and determine the severity of sarcopenia [5].

The identification of sarcopenia cases is associated with the use of the 5-item SARC-F questionnaire. With results self-reported by subjects, the questionnaire can be utilized as a screening tool for the assessment of sarcopenia hazards [5,6]. SARC-F has a low-to-moderate sensitivity and a very high specificity in terms of foreseeing low muscle strength. As a result, its use will mainly expose severe sarcopenia cases [5]. Apart from this test, other tests such as anthropometric measures, Mini Sarcopenia Risk Assessment (MSRA) Questionnaire, Ishii Test, Taiwan Risk Score for Sarcopenia, Sarcopenia Scoring Assessment Model (SarSA-Mod), Sarcopenia Quality of Life (SARQoL) questionnaire, and the fracture risk assessment tool have been examined as probable screening tools for sarcopenia [6,7,8].

Measuring muscle strength by evaluating grip strength is quite manageable and low-cost [5,9]. It is already well-established that low grip strength is a significant predictor of poor outcomes including increased hospitalizations and functional difficulties, as well as poor health-related quality of life (QoL) with increased mortality [5,10]. The accurate assessment of grip strength is obtained via the utilization of a calibrated handheld grip-strength dynamometer [5,11], in which the cut-off points concerning men and women related to low grip strength and probable sarcopenia are <27 kg and <16 kg, respectively [5]. In addition, the “chair stand test”, which is also named the “chair rise test”, might be utilized as a representative measure assessing the strength of leg muscles (quadriceps muscle group) [5,12]. The chair stand test evaluates the sum of time required for a subject to rise five times from a seated position without utilizing his or her arms [5].

Muscle quantity or mass can be evaluated using various techniques, and there are different means of adjusting the outcome for body mass index (BMI) or height [5]. Muscle quantity could be recorded as appendicular skeletal muscle mass (ASM), as total body skeletal muscle mass (SMM), or as muscle cross-sectional area of specific muscle groups or body locations [5]. It is already well-established that magnetic resonance imaging (MRI) and computed tomography (CT) are gold-standard methods for the non-invasive evaluation of muscle quantity/mass, permitting the assessment of muscle quality and fatty infiltration. However, their utilization is so far mainly associated with research [5,13,14]. Moreover, dual-energy X-ray absorptiometry (DXA) can be characterized as a more broadly available means of assessing muscle quantity non-invasively. Additionally, it is the sole radiological tool with accepted cutoff values to identify sarcopenia [5,14,15]. While bioelectrical impedance analysis (BIA) does not evaluate muscle mass directly, it alternately provides an estimation of muscle mass that depends on whole-body electrical conductivity [5,16].

Concerning physical performance, it can be evaluated using gait speed, the short physical performance battery (SPPB), and the timed up and go test (TUG) [5,17,18].

Other tests associated with the assessment of sarcopenia might include lumbar 3rd (L3) vertebra imaging via CT, mid-thigh muscle imaging (using MRI or CT), psoas muscle measurement using CT, and ultrasound (U/S) evaluation of the muscle [5].

At this moment, resistance exercise (RE) is suggested as a first-line therapy to confront the adverse outcomes of sarcopenia [19]. Even though a low-intensity resistance training program is adequate in terms of providing strength gains, it has been recorded that a high-intensity resistance training strategy could be beneficial to obtaining maximal strength gains [20]. In addition, multimodal exercises and blood flow restriction resistance training could be advisable [20].

Concerning nutrition in sarcopenic subjects, several trials have demonstrated that dietary interventions, including protein intake, improve functional and/or strength outcomes, whilst it seems that different dietary interventions are not quite as fruitful [21,22]. Both starvation and aggressive hypocaloric diets have been recorded as being detrimental to the skeletal muscle mass and muscle function, particularly when protein needs are not met [23]. That is probably owing to the inhibition of the mammalian target of the rapamycin complex 1 (mTORC1) pathway, as shown after some weeks of low-carbohydrate high-fat (LCHF) diets [23].

Loop diuretics (LDs) are basically the first-line-administered medication in the management of hypervolemia, with additional medication classes indicated in clinical cases of diuretic resistance and electrolyte or acid–base disarrangements [24].

LDs inhibit Na+ (and consequently water) resorption from the ascending limb of the loop of Henlé in the renal tubule [25]. Moreover, they augment the urinary excretion of K^+^, Mg^2+^, H^+^ and CI^–^. Meanwhile, LDs, of which furosemide is the most frequently administrated, are utilized to manage fluid overload in heart failure (HF), renal disease (RD), liver cirrhosis and hypertension in order to improve manifestations of breathlessness and edema [25,26]. Nevertheless, a diuretic-induced reduction in plasma volume might activate several neurohumoral systems, such as renin–aldosterone–angiotensin, leading to impaired renal perfusion and increased Na^+^ and water resorption [25]. These alterations contribute towards a decreased diuretic, which is called diuretic resistance or also renal function impairment [25].

Furosemide (frusemide) is a frequently utilized diuretic that belongs to LDs. It is utilized in the treatment of oedematous conditions related to renal, cardiac and hepatic failure, and for the management of hypertension [27]. Other LDs are bumetanide and torsemide (rINN torasemide). While these may be more expensive in comparison with furosemide, they have a higher (≥80%) and more consistent per os (PO) bioavailability [25]. This might mean that some subjects might have a greater diuresis when switched to them from furosemide [25].

LDs are related to various adverse outcomes such as hypokalemia, ototoxicity, and others [26]. LDs can potentially interact with other medications. It is already established that LDs might interact with several medications including digoxin, amphotericin B, angiotensin-converting enzyme inhibitors (ACE inhibitors), antifungal agents, antidiabetic drugs, dobutamine, and sotalol due to diuretic-associated hypokalemia [26]. Moreover, the hazard of ototoxicity could be augmented by the concomitant utilization of LDs, cisplatin, aminoglycoside antibiotics or phosphodiesterase 5 (PDE 5) inhibitors [26]. Moreover, LDs might interact pharmacodynamically with medications such as levothyroxine, cephalosporins, pixantrone, ceritinib, lithium, probenecid, nonsteroidal anti-inflammatory drugs (NSAIDs), sulfonylureas, and other herbal drugs [26].

It is already recorded that polypharmacy is associated with sarcopenia or the risk of sarcopenia [28]. In this article, we attempted to examine the impact of a specific medication category such as LDs on sarcopenia and skeletal muscle mass health by thoroughly examining the existing current literature.

## 2. Materials and Methods

**Figure 1:**
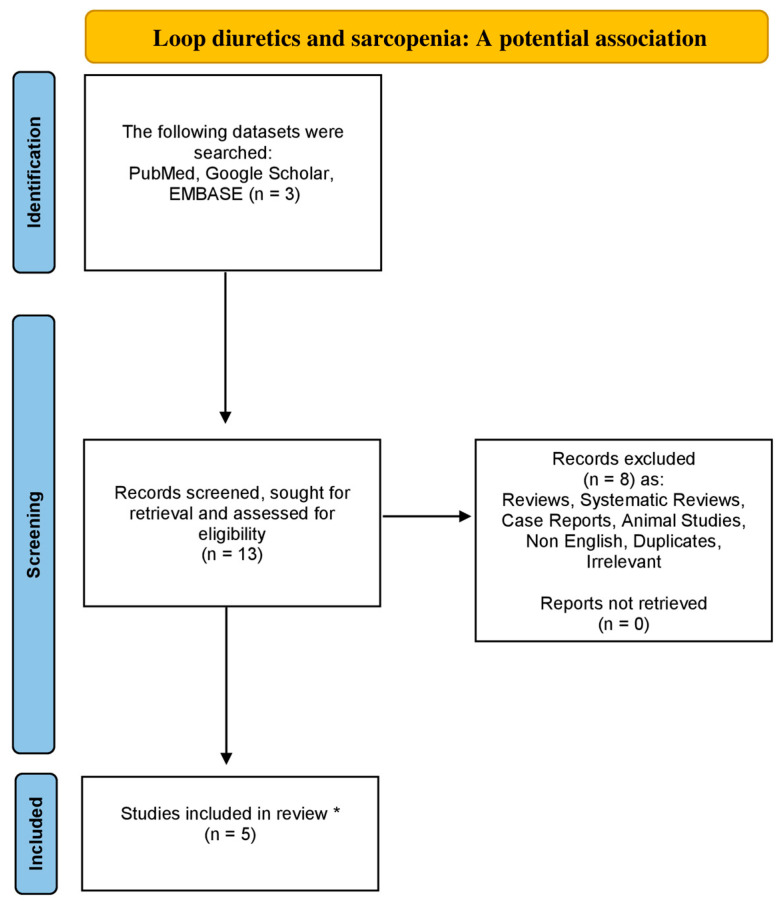
Flowchart diagram indicative of the literature review organization concerning sarcopenia and loop diuretics association (* only original, English and non-animal studies were included in this non-systematic review article).

Utilizing the databases of Google Scholar, EMBASE and PubMed from 1984 to August 2023, using the following combinations of particular keywords: “sarcopenia” or “low muscle mass” and “loop diuretics”, we conducted the research for a non-systematic review article. Original articles written in the English language were included in this review study. Moreover, all the references concerning the included studies were also exhaustively examined. Studies related to animals were excluded. Our strategy is demonstrated in the flowchart diagram (Figure 1).

## 3. Results

The basic aim of this article was to demonstrate the existence of any probable association between LDs and sarcopenia, as recorded by the existing literature. The outcomes of this non-systematic review study are depicted in Table 1.

Ishikawa et al. attempted to investigate and demonstrate the prevalence and risk factors for sarcopenia among subjects with non-dialysis-dependent chronic kidney disease (NDD-CKD) and they specifically focused on the utilization of medications [29]. They conducted a cross-sectional study including a cohort of 260 subjects with NDD-CKD, recruited between June 2016 and March 2017, during which they recorded the appropriate data concerning gender, age, cause of chronic kidney disease, utilization of drugs, and comorbidities that might potentially have an impact on sarcopenia [29]. The sarcopenia assessment and evaluation were conducted using the criteria of the Asian Working Group for Sarcopenia (AWGS), while logistic regression analysis was performed in order to clarify the relation of each factor on the prevalence of sarcopenia [29]. Their intriguing results demonstrated that 25.0% of their study patients had sarcopenia, while the multivariable analysis showed that an increased risk of sarcopenia was importantly related to age, male gender, diabetes mellitus, body mass index (BMI), and LD utilization (odds ratio [OR], 4.59; 95% confidence interval [CI], 1.81–11.61; *p* = 0.001) [29]. They concluded that the prevalence of sarcopenia in subjects with NDD-CKD was increased. Additionally, diuretic utilization, especially LD utilization, was demonstrated to be a risk factor of sarcopenia [29].

Hanai et al. aimed to examine the effect of LDs that are usually utilized to confront ascites/hepatic edema on skeletal muscle depletion and the prognosis of subjects living with liver cirrhosis [30]. In their retrospective study, they evaluated 226 subjects living with liver cirrhosis. Then, the skeletal muscle cross-sectional area at the level of the third lumbar vertebra was studied utilizing CT imaging [30]. In a median follow-up period of 49 months, 82 subjects passed away [30]. The relative change in skeletal muscle area per year (ΔSMA) was calculated, and the relation between ΔSMA and the therapeutic dosage of LDs was evaluated [30]. Their results demonstrated that the therapeutic dosage of LDs was inversely correlated with ΔSMA when subjected to simple (r = −0.27, *p* < 0.0001) and multiple regression analyses (t = −3.07, *p* = 0.002). Moreover, this study showed that overall survival rates were lower in subjects with LDs at >20 mg than those at ≤20 mg (median, 66 vs. 97 months; *p* = 0.002), while the multivariate analysis showed that LDs of >20 mg (hazard ratio [HR], 1.86; 95% CI, 1.03–3.24; *p* = 0.039) and ΔSMA of ≤−3.1% (HR, 3.87; 95% CI, 2.32–6.60; *p* < 0.0001) were independently associated with mortality [30]. They concluded that a higher dose of LDs utilization was linked to a quicker skeletal muscle depletion and poor prognosis in subjects living with liver cirrhosis, independent of the liver disease severity [30].

In another study, Nakano et al. tried to investigate the impact of LDs on skeletal muscle mass of subjects living with HF [31]. In their subanalysis of a cross-sectional study from 10 hospitals, assessing 155 patients with HF (age 67 ± 13 yrs, 69% men), they compared the HF subjects treated with LDs (n = 120) with the subjects who had not undergone this treatment (n = 35) [31]. They demonstrated that both the thigh and arm circumferences were significantly smaller in the group of subjects treated with LDs in comparison with those not treated in this way (39.9 ± 4.8 vs. 43.5 ± 6.9 cm; *p* < 0.001 and 26.7 ± 3.5 vs. 28.9 ± 6.2 cm; *p* < 0.001, respectively) [31]. Moreover, in the univariate analysis, higher age, lower hemoglobin, lower BMI, and LDs utilization were importantly related to smaller thigh circumference. Conversely, in multivariable analysis, the utilization of LDs was independently related to a smaller thigh circumference (β = −0.51; 95% CI −0.98 to −0.046; *p* = 0.032) [31]. The authors concluded that LDs are related to reduced thigh and arm circumferences in subjects with HF, independent of the severity of HF, revealing the adverse impact of LDs on skeletal muscle wasting [31].

Another study conducted by Saitoh et al. tried to investigate and evaluate determinants of anorexia which is loss of appetite in subjects with HF and aimed to further demonstrate the relation between anorexia, functional capacity, and clinical outcomes [32]. The authors evaluated anorexia status among 166 subjects with HF (25 female; 66 ± 12 years) and anorexia was evaluated using a 6-point Likert scale (ranging from 0 to 5), wherein values ≥1 indicated anorexia, while a number of 22 subjects (13%) passed away during a mean follow-up of 22.5 ± 5.1 months [32]. Functional capacity was evaluated as the results of peak oxygen uptake (peak VO_2_), a 6 min walk test, and a short physical performance battery test [32]. In total, 57 subjects (34%) reported any anorexia, and these subjects demonstrated lower values of peak VO_2_, 6 min walk distance, and short physical performance battery scores (all, *p* < 0.05) [32]. Multivariate analysis, adjusting for clinically significant factors, demonstrated that only high-sensitivity C-reactive protein (OR, 1.24; *p* = 0.04], the utilization of LDs (OR, 5.76; *p* = 0.03), and the presence of cachexia (OR, 2.53; *p* = 0.04) are independent predictors of anorexia [32]. In addition, Kaplan–Meier curves for cumulative survival demonstrated that those subjects with anorexia presented increased mortality (log-rank test, *p* = 0.03) [32]. In comparison with subjects without anorexia, anorexic subjects were more likely to be on diuretic drugs (75.5% vs. 96.5%; *p* = 0.001) and utilize LDs (56.9% vs. 78.9%; *p* = 0.006), while the attendance of sarcopenia was not importantly different between anorexic and non-anorexic subjects. However, those with from both anorexia and cachexia demonstrated increased prevalence of muscle wasting (subjects having both anorexia and cachexia—40%; anorexia—13.9%, cachexia—10.0%; and neither anorexia nor cachexia—11.2%) [32].

On the contrary, a study conducted by Sawada et al. tried to evaluate the impact of diuretics and skeletal muscle depletion on muscle cramps utilizing a questionnaire survey [33]. They enrolled 152 subjects (mean age, 68.5 ± 11.5 years; 62.5% male; BMI, 24.3 ± 3.7) with liver cirrhosis. The main causes of cirrhosis were viral hepatitis type B in 13%, viral hepatitis type C in 41%, nonalcoholic fatty liver disease in 7%, alcoholic liver disease in 30%, autoimmune hepatitis and primary biliary cholangitis in 5%, and others in 4% of cases [33]. Among the 78 subjects (51%) who sustained muscle cramps in the preceding 12 weeks, the frequency of muscle cramps was recorded as several times per month (55%), several times per week (33%), and several times per day (12%). Cramp questionnaires were recorded after receiving informed consent, while body composition, including muscle volume, was evaluated utilizing a bioelectrical impedance analysis (BIA) method. Muscle strength (hand grip strength) was also assessed. In addition, the cross-sectional skeletal muscle area was assessed using CT imaging at the L3 vertebral level in order to further study the association between muscle cramps and sarcopenia [33]. The authors divided subjects into those with or without muscle cramps. Interestingly, body composition, including grip strength and presence of sarcopenia (*p* = 0.73), was similar across the 2 groups [33]. As for diuretics utilization, furosemide utilization was significantly higher in the muscle cramp group (24% vs. 9.5%; *p* = 0.01), while multivariate logistic regression analysis demonstrated that furosemide utilization (OR, 2.61; 95% CI, 1.01–6.78; *p* = 0.04) was the sole noteworthy predictor for muscle cramp occurrence [33].

## 4. Discussion

In this article, we aimed to examine the newly relevant interplay between the use of LDs and sarcopenia. It seems that the use of this specific drug category has an effect on skeletal muscle mass health and might be negatively associated with sarcopenia, leading to probable adverse outcomes concerning public health that should raise awareness among physicians and laboratory scientists who investigate these potential effects.

There are specific limitations associated with this non-systematic review article. The number of studies available in the existing current literature is quite scarce, while the number of patients participating in those that already exist is limited. In addition, the follow-up periods of participating subjects are mainly small for most of the studies. Nonetheless, it would be quite interesting and intriguing if more and different types of studies with larger numbers of participating subjects under LDs could be conducted in the near future. These could derive from various medical centers all around the world and enroll different types of population with varied medical histories and LD-treated diseases.

Another intriguing approach associated with this health issue and the probable feedback between these two entities could be the investigation of other LDs and their impact on sarcopenia and skeletal muscle health, with the exception of furosemide. A significant concept worth considering would be the investigation of the potential impact of diuretic combinations on skeletal muscle health and sarcopenia or the identification of the optimum diuretic medication for sarcopenic subjects in need of diuresis. In addition, it would be significant if we could discover more about the minimum dosage of each LD required in the treatment of various and different long-term health issues that could trigger or impair sarcopenia in order to intercept any potential adverse result. The validation of an index or score that could relate the LD dosage with the risk of sarcopenia would be a significant step towards prevention.

Further investigation concerning specific nutritional programs and interventions in subjects under LD treatment would be of great importance in preventing sarcopenia or fortifying subjects who are already sarcopenic. Moreover, these nutritional interventions should take under serious consideration the main disease of the subjects treated with LDs because many diseases have already been potentially associated with sarcopenia and adverse outcomes towards skeletal muscle health [34,35,36,37,38]. The collaboration between clinical nutritionists and physicians should be an important step in that direction.

Last but not least, appropriate and specific training programs should also be studied and proposed in sarcopenic patients or subjects treated with LDs at risk of sarcopenia in order to enhance and fortify their skeletal muscle mass health. These physical training programs should also consider the basic medical condition treated with LDs and the ability of each subject to adjust to the selected physical training program. Cooperation between physical trainers and physicians seems to be imperative in the near future to assure the optimum approach. After all, assessing sarcopenia is on many occasions quite intriguing.

## 5. Conclusions

Studying thoroughly the existing current literature concerning the two under investigation abovementioned entities, we demonstrated a potential and upcoming interplay between LDs utilization and sarcopenia. Particularly, we found that, in subjects with NDD-CKD, increased risk of sarcopenia is significantly related to LD use. Interestingly, in subjects with HF treated with LDs, thigh and arm circumferences were found to be significantly small. Additionally, a higher prevalence of sarcopenia was demonstrated in anorexic subjects who are more likely to be on diuretic medication, suffering also from cachexia, while in cirrhotic subjects the therapeutic dosage of LDs was inversely correlated with ΔSMA. Nevertheless, after their division into two groups, including those with and without muscle cramps, the presence of sarcopenia was found to be similar in subjects living with liver cirrhosis undergoing treatment with LDs. Further studies are needed to provide sufficient data and validate these claims. Moreover, the collaboration of medical specialists with clinical dieticians and physical trainers should be widely applied in order to provide a more personalized approach.

## Figures and Tables

**Table 1:** The association between loop diuretics and sarcopenia.

Authors/[Reference]	Study Design	Study Population	Main Outcomes	Sarcopenia Assessment
Ishikawa et al./[29]	Cross-sectional	A total of 260 subjects with NDD-CKD.	In total, 25.0% of subjects had sarcopenia. Increased risk of sarcopenia was importantly related to age, male gender, DM, BMI, LD use (OR, 4.59; 95% CI, 1.81–11.61; *p* = 0.001).	AWGS
Hanai et al./[30]	Retrospective	A total of 226 liver cirrhosis subjects. During follow-up period, 82 subjects died.	Therapeutic dosage of LDs inversely correlated with ΔSMA when conducted using simple (r = −0.27; *p* < 0.0001) and multiple regression analyses (t = −3.07; *p* = 0.002). Overall, survival rates decreased more in subjects treated with LDs at >20 mg than those treated at ≤20 mg (median, 66 vs. 97 months; *p* = 0.002). LDs of >20 mg (HR, 1.86; 95% CI, 1.03–3.24; *p* = 0.039) and ΔSMA of ≤−3.1% (HR, 3.87; 95% CI, 2.32–6.60; *p* < 0.0001) independently related to mortality.	Skeletal muscle cross-sectional area at the level of L3 vertebra measured using CT.
Nakano et al./[31]	Cross-sectional	A total of 155 subjects with HF (age 67 ± 13 years, 69% men).	Thigh and arm circumferences were significantly small in the LD-treated group in comparison with those not treated (39.9 ± 4.8 vs. 43.5 ± 6.9 cm; *p* < 0.001 and 26.7 ± 3.5 vs. 28.9 ± 6.2 cm; *p* < 0.001, respectively). LDs were significantly related to smaller thigh circumference and independently related to smaller thigh circumference (β = −0.51; 95% CI −0.98 to −0.046; *p* = 0.032).	Thigh and arm circumferences
Saitoh et al./[32]	Prospective, observational	A total of 166 subjects with HF (25 female; 66 ± 12 years). 22 subjects (13%) died during follow-up.	Anorexic subjects are more likely to be on diuretic treatment (75.5% vs. 96.5%; *p* = 0.001) and LDs utilization (56.9% vs. 78.9%; *p* = 0.006). Subjects having both anorexia and cachexia demonstrated increased prevalence of sarcopenia.	DXA
Sawada et al./[33]	Cross-sectional questionnaire	A total of 152 subjects (mean age, 68.5 ± 11.5 years; 62.5% male; BMI, 24.3 ± 3.7) with liver cirrhosis	Two groups including subjects with or without muscle cramps. Body composition, grip strength and sarcopenia (*p* = 0.73) were similar in the 2 groups. Furosemide use was significantly higher in the muscle cramp group (24% vs. 9.5%; *p* = 0.01), while furosemide utilization (OR, 2.61; 95% CI, 1.01–6.78; *p* = 0.04) was the sole noteworthy predictor of muscle cramp occurrence.	CT imaging at the L3 vertebral level, hand grip strength and BIA

Abbreviations: NDD-CKD: non-dialysis-dependent chronic kidney disease; DM: diabetes mellitus; BMI: body mass index; LDs: loop diuretics; OR: odds ration; CI: confidence interval; AWGS: Asian working group for sarcopenia; ΔSMA: skeletal muscle area per year; HR: hazard ratio; L3: third lumbar; CT: computed tomography; HF: heart failure; DXA: dual-energy X-ray absorptiometry; BIA: bioelectrical impedance analysis.

## Data Availability

Not applicable.
